# *Entamoeba dispar*: A Rare Case of Enteritis in a Patient Living in a Nonendemic Area

**DOI:** 10.1155/2014/498058

**Published:** 2014-02-13

**Authors:** Rosalia Graffeo, Carola Maria Archibusacci, Silvia Soldini, Lucio Romano, Luca Masucci

**Affiliations:** Institute of Microbiology, Catholic University of the Sacred Heart, Policlinico “Agostino Gemelli”, Largo Agostino Gemelli, 8-00168 Rome, Italy

## Abstract

*Entamoeba dispar*, a common noninvasive parasite, is indistinguishable in its cysts and trophozoite forms from *Entamoeba histolytica*, the cause of invasive amebiasis, by microscopy. To differentiate the two species seems to be a problem for laboratory diagnosis. Recent experimental studies showed that  *E. dispar* can be considered pathogenic too. We present a rare case of enteritis due to *E. dispar*.

## 1. Introduction

*Entamoeba histolytica *and *Entamoeba dispar *are two distinct but morphologically identical species living in the human colon [1, 2].

*E. histolytica* causes amebiasis. Amebiasis is one of the most common causes of death from protozoan parasitic disease, second only to malaria, with approximately 50 million cases and 100 000 deaths annually, as reported by the WHO [3] and in areas where invasive amebiasis is common; *E. dispar *is by far the more prevalent species [4].

Until a few years ago, several studies distinguished between infections caused by *E. histolytica*, with invasive intestinal and extraintestinal disease, and those by *E. dispar *and* E. moshkovskii*, which were not considered pathogenic [5].

Recent studies showed *E. dispar *trophozoites to produce focal lesions in experimental animal models and to have lytic activity in cultured monolayer epithelial cells [6].

In 2012 Dolabella et al. described* E. dispar* trophozoites from the ICB-ADO strain (zymodeme I-nonpathogenic), inoculated intrahepatically in hamsters, which produced amoebic liver abscess [7].

Antigen detection, culture, and polymerase chain reaction are employed to distinguish *E. histolytica* from *E. dispar*. We report a case of enteritis due to *E. dispar*.

## 2. Case Report

An Italian 81-year-old woman suffered of abdominal pain and chronic diarrhea. She evacuated unformed stools three times a day for 10 days. She had not been hospitalized in the last year nor had she traveled to tropical countries.

Blood biochemistry and liver function tests were normal, and she was serologically negative for human immunodeficiency virus (HIV). Multiple stool cultures for bacterial pathogens, including *Salmonella*, *Shigella*, and *Campylobacter*, enterotoxigenic and other pathogenic *E. coli *and *C. difficile* toxin A/B were negative.

Stools collected for parasites were negative for ova and larvae by microscopy and for *Giardia intestinalis* and *Cryptosporidium parvum* by immunochromatographic test (CerTest Biotec S.L. Zaragoza—Spain).

**Figure 1:**
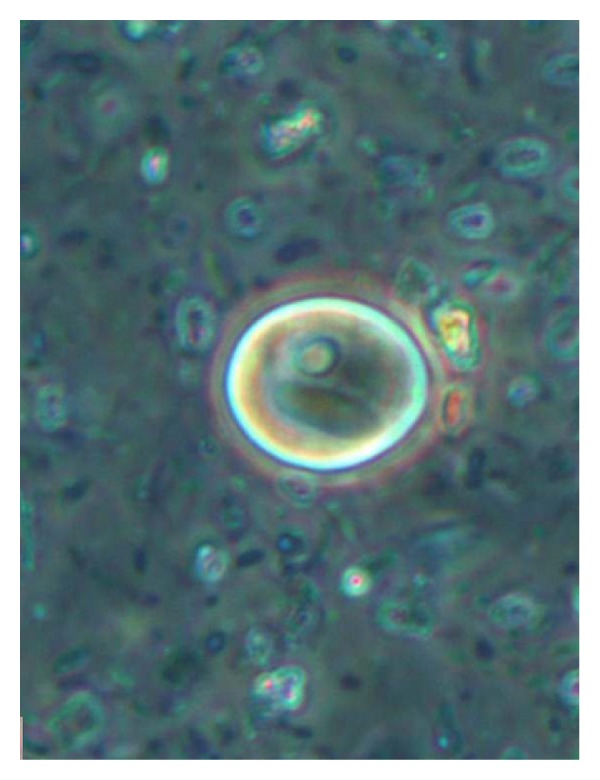
*Entamoeba histolytica/dispar/moshkovskii *cyst (original magnification 400x).

*Entamoeba histolytica/dispar/moshkovskii* cysts were detected by microscopy at wet smear preparation with a 400x phase-contrast objective (Figure 1).

Culture was performed in medium based on Boeck and Drbohlav formulation (DiaSys Entamoeba kit, DiaSys Europe Ltd; Wokingham, UK).

We also used DNA-based methods to speciate *E. histolytica*, *E. dispar*, and *E. moshkovskii* as previously described [8–10].

DNA was extracted from the clinical sample using the QIAamp DNA Mini Kit (Qiagen) according to manufacturer's instructions.

The nested multiplex polymerase chain reaction (PCR) [10], performed on stool, showed the species-specific product size for *E. dispar *(174 bp).

**Figure 2:**
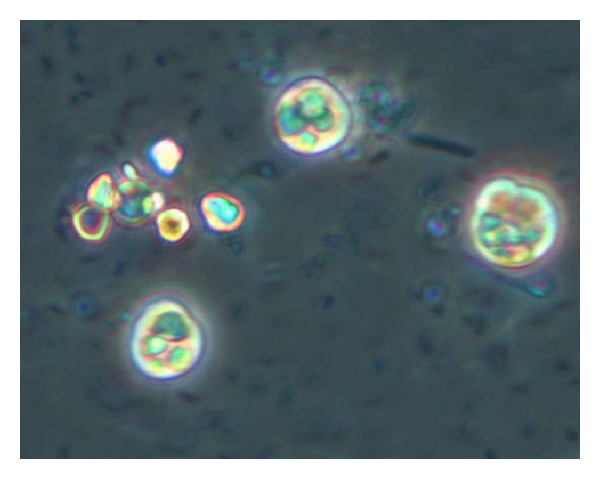
*Entamoeba dispar *trophozoites (molecular typing) in Boeck and Drbohlav formulation medium (original magnification 400x).

After 2 days, typical *E. histolytica* trophozoites were visualized in culture by microscopy (Figure 2).

PCR was performed on culture using two more protocols [8, 9].

By these methods, PCR products were specific for *E. dispar *too. The DNA amplicons were analyzed using the sequencing kit ABI PRISM dye terminator cycle and the automatic sequencing system ABI 3100 (Applied Biosystems), confirming *E. dispar *(GenBank accession number AB282661.1, genes for 18S rRNA, ITS1, 5.8S rRNA, ITS2).

We established a treatment protocol the same as luminal amebiasis (Paromomycin 30 mg/kg/day for 10 days in 3 divided doses). Patient responded promptly with resolution of diarrhea in 2 days.

Thirty days later, we used microscopy and PCR to test six samples of stool collected over a fourteen-day period. All samples were negative for *E. histolytica/dispar*.

## 3. Conclusion

Microscopy is still the gold standard to detect *Entamoeba spp*. in stool samples, but the use of routine diagnostic methods such as concentration and trichrome staining may be insufficient to demonstrate the presence of *E. histolytica/dispar* [11]. Studies about fecal antigen detection for pathogenic *E. histolytica *provide sensitivities and specificities that range from 87 to 97.6% and 92.6 to 98%, respectively [11]. However PCR-based methods revealed greater sensitivity and specificity than ELISA (enzyme-linked immunosorbent assay) stool antigen detection kits, which employ monoclonal antibodies for the detection of *E. histolytica *and *E. histolytica/dispar* and that cannot discriminate between these genetically distinct organisms [12].

To distinguish *E. histolytica* and *E. dispar*, various molecular methods can be used [8–10].

Cultivation of *E. histolytica* plays a minor role, because this test is difficult, expensive, and labor-intensive to be routinely used in the diagnostic laboratory [13].

Culture techniques for *Entamoeba spp*. include xenic (diphasic and monophasic) and axenic systems. Culture of *E. dispar* is reported, but only in axenic or monoxenic medium [14, 15]; protozoa isolated from the patient in this study has grown in a xenic culture.

Xenic cultivation is defined as the growth of the parasite in the presence of an undefined bacteria flora, while monoxenic culture occurs in the presence of a specific bacterium. The xenic culture of *E. histolytica *was first introduced by Boeck and Drbohlav in 1925 in a diphasic egg slant medium, and a modification of this medium (Locke-egg) is still used today. Purified rice starch is important for growth of *E. histolytica *as a carbohydrate source in xenic cultivation. In fact soluble sugars cannot be used because they would be metabolized rapidly by the bacteria [13]. Within 1–4 days of incubation at 37°C in this medium, *E. histolytica *trophozoites appear with characteristic hyaline ectoplasm with engulfed starch crystal, according to manufacturer's instructions.

*E. dispar* in humans was always considered a commensal organism. Using an animal model, it has also been demonstrated that this species causes tissue lesions in the intestine and serious damage to epithelial cells [16, 17].

The spectrum of clinical manifestations observed widely varies among individuals infected with *E. dispar*, ranging from amoebic liver abscess production [18] to symptomatic nondysenteric colitis [6]. Some authors even recommend not to treat the latter infection [19].

The patient observed in this study was investigated for bacterial pathogens and parasites. Stool samples analyzed were positive only for *E. dispar*. In this case, therapy was decisive, so the patient could completely recover.

Other intestinal parasites such as *Cyclospora cayetanensis* were always considered pathogens for humans in endemic areas and linked to travellers and immunocompromised patients, but cases of immunocompetents and no-travel relations are reported [20]. Infections due to protozoa are acquired by the fecal-oral route through ingestion of infective oocysts or cysts in contaminated water or on contaminated fruits and vegetables, which today, in the “globalization era,” is easy to observe everywhere [21].

Like in this report, diseases and pathogens are constantly evolving and for this reason to the question of Ximénez et al. “Human amebiasis: breaking the paradigm?” we answer “YES.”
